# Development and Evaluation of pH-Dependent Micro Beads for Colon Targeting

**DOI:** 10.4103/0250-474X.62230

**Published:** 2010

**Authors:** M. S. Khan, B. K. Sridhar, A. Srinatha

**Affiliations:** Department of Pharmaceutics, National College of Pharmacy, Shivamogga-577 201, India

**Keywords:** Alginate, chitosan, colon targeting, Eudragit S100, multiparticulates, theophylline

## Abstract

The purpose of this research was to develop and evaluate multiparticulates of alginate and chitosan hydrogel beads exploiting pH sensitive property for colon-targeted delivery of theophylline. Alginate and chitosan beads were prepared by ionotropic gelation method followed by enteric coating with Eudragit S100. All formulations were evaluated for particle size, encapsulation efficiency, swellability and *in vitro* drug release.*In vitro* dissolution studies performed following pH progression method demonstrated that the drug release from coated beads depends on coat weights applied and pH of dissolution media. Mechanism of drug release was found to be swelling and erosion-dependent. The studies showed that formulated alginate and chitosan beads can be used effectively for the delivery of drug to colon and a coat weight of 20% weight gain was sufficient to impart an excellent gastro resistant property to the beads for effective release of drug at higher pH values.

There has been a considerable research for design of colonic drug delivery system. Colon targeting has been achieved by several ways, which include prodrugs, pH- and time-dependent systems. Polysaccharides such as chitosan, pectin, inulin, alginate, guar gum have been explored for their potential for colon specific drug delivery. To achieve successful colon drug delivery, a drug needs to be protected from degradation, release and/or absorption in upper portion of GI tract and then ensure abrupt or controlled release in proximal colon. Colon targeting is usually advised because of advantages i.e, reduced dosing frequency; achieve high concentration at distal gut for local treatment; chronotherapy and delivery of drug to a region that is less hostile metabolically[1].

Multiparticulates offer greater advantages over single unit system as they disperse uniformly in GI tract, offer flexibility and less inter and intra individual variability in formulation process. Multiparticulates present several advantages in comparison to single unit forms that they exhibit higher colonic residence time and more predictable gastric emptying[2].

Alginate is linear, naturally occurring polysaccharide extracted from brown sea algae. In presence of various divalent (Ca^2+^ and Zn^2+^) or trivalent ions (Al^3+^), an elastic gel is formed due to ionic interaction between the ions and carboxyl group, mainly of guluronic blocks. Chitosan, a naturally occurring polysaccharide has received major attention in drug delivery system. It is biocompatible and non toxic. Chitosan is weak cationic polysaccharide composed of (a (1→4) 2-amino-2deoxy-β-D-glucan) obtained by alkaline deacetylaion of chitin[3].

Asthma is a chronic obstructive lung disease characterized by airways inflammation and hyper reactivity. In most patients, conditions worsen at night with acute exacerbation being in most common[4]. Clinical and epidemiological studies suggest that asthma is several hundred folds more likely at night than during day with disturbance of sleep. The heightened risk of asthma at night coincides with the trough of circadian rhythm. A colonic drug delivery system would be valuable when a delay in absorption of drug is therapeutically desirable in treatment of chronic conditions like nocturnal asthma, which coincides with trough of circadian rhythm[5].

The physiological changes in pH of gastrointestinal tract have been extensively exploited to target the actives moieties to colon. Methods based on pH sensitive delivery such as delayed onset dosage forms could be a simple and practical means for colon targeting. Polymer particularly Eudragit S100 have been investigated for colonic delivery. Since the pH of colon in normal subjects varies from 6.4±0.6 to 7.5±0.4, these polymers have been designed to be soluble at pH >7[6].

The aim of present investigation was to work out feasibility of combination of multiparticulates and pan coating to produce pH sensitive micro beads of theophylline for colon targeting. The major objective was to modulate drug release from these coated multiparticulates to specifically target the nocturnal peak symptoms of asthma.

## MATERIALS AND METHODS

Theophylline hydrochloride (TH) was a gift sample from French Pharma, Chandigarh, India. Chitosan (CH) and sodium alginate were purchased from Sigma Aldrich Chemicals, Bangalore, India. Triethyl citrate was purchased from Himedia laboratories Pvt. Ltd, Mumbai. Ethylcellulose (EC) was purchased from S. D. Fine-Chem Limited, Mumbai, India. Eudragit S100 was a gift sample from Cadila, Ahmedabad, India. All other chemicals used were of analytical reagent grade.

### Preparation of drug loaded beads:

Alginate, chitosan beads containing theophylline was prepared by ionotropic gelation method. Theophylline was dispersed in sodium alginate (2% w/v) solution or chitosan (3% w/v) solution in suitable solvents (Table 1). The drug-polymer dispersion was dropped through a needle (#20) into agitated 5% w/v aqueous calcium chloride solution (100 ml) and 2% w/v aqueous solution of tripolyphosphate (pH adjusted to 5), respectively for alginate and chitosan. Beads were left for 30 min, and washed twice with distilled water and dried at 50° for 4 h and then at room temperature (25°) for 2 days for complete drying[7–9].

**TABLE 1:** FORMULATIONS VARIABLES OF PREPARED HYDROGEL BEADS

Type of Beads	Formulation	Drug:Polymer	Hardening solution (% w/v)
Alginate	A1	1:2	Calcium chloride
Alginate	A2	1:3	Calcium chloride
Alginate	A3	1:4	Calcium chloride
Ethylcellulose reinforced beads	A4	1:2	Calcium chloride
Chitosan reinforced beads	A5	1:3	Calcium chloride with chitosan
Chitosan	C1	1:1	Tripolyphosphate
Chitosan	C2	1:1.5	Tripolyphosphate
Chitosan	C3	1:2	Tripolyphosphate
Chitosan	C4	1:3	Tripolyphosphate

### Polymer reinforced beads:

Theophylline was dispersed uniformly in sodium alginate (2% w/v) solution and ethylcellulose (2% w/v) was mixed and stirred for 30 min and then drop wise added to agitated 5% w/v calcium chloride solution (100 ml). Chitosan reinforced alginate beads were prepared by dispersing theophylline in sodium alginate solution (2% w/v). The solution was dropped through a needle (#20) into agitated 5% calcium chloride solutions (100 ml) with chitosan (0.5% w/v) already dissolved in acetic acid solution (1% v/v). Beads were left for 30 min, and washed twice with distilled water and dried at 50° for 4 h and then at room temperature (25°) for 2 days for complete drying[10,11].

### Coating of beads:

The enteric coating of all the beads bearing theophylline was performed by spray coating in a conventional coating pan. In brief coating solution was prepared by mixing Eudragit S100 with acetone for 1 h using a stirrer. After an hour, triethyl citrate (TEC) 6*%* w/v was added and stirring was continued for 30 min. Coating was done with a pan rotating at 20 rpm, with an inlet air temperature 60 to 70° and outlet air temperature of 35 to 40°. The solution was sprayed at an atomizing air pressure of 2 bars. The coating was continued until the desired weight gain was achieved. A series of coated products with different film thickness were produced, quantified by the total weight gain (%TWG), by varying amount of coating solution sprayed[12].

### Particle size analysis:

The particle size of prepared beads was measured with an optical microscope fitted with a calibrated eyepiece. The mean of 50 beads was noted as particle size. To determine the particle size of ethyl cellulose reinforced alginate beads dial thickness meter was used.

### Drug content and encapsulation efficiency:

The theophylline in the beads was determined by digesting the beads in PBS (pH 7.4) and 0.1 N HCl (pH 1.2), respectively, for sodium alginate and chitosan beads. The extracted drug concentration was determined spectrophotometrically (UV-1601, Shimadzu, Japan) at 272 nm for alginate beads and 269 nm for chitosan beads. The encapsulation efficiency of prepared hydrogel beads was calculated in terms of ratio of drug in formulation to amount of drug added[5].

### Swelling studies:

Swelling studies for coated beads was performed in three different media simulating transition of beads in GIT (pH 1.2, 6.8 and 7.4). Swelling index was calculated from the formula: Swelling index= (W_g_–W_o_)/Wo×100, where W_o_ is the initial weight of beads and W_g_ is the weight of beads in the swelling medium[12–14].

### *In vitro* drug release studies:

*In vitro* drug release studies were performed using USP dissolution test apparatus (Apparatus 2). The dissolution studies were performed in 900 ml of dissolution medium, which was stirred at 100 rpm at 37±0.1° following a pH progression method i.e pH 1.2 for first 2 h, pH 6.8 for next 3 h and pH 7.4 for rest of studies. Aliquots was withdrawn periodically and replaced with fresh medium. Theophylline content in the aliquots was assayed spectrophotometrically (UV-1601, Shimadzu, Japan) at 269 nm for pH 1.2 and 272 nm for the rest of the samples[15].

## RESULTS AND DISCUSSION

Alginate and chitosan beads were prepared by dropping the solution of the drug alginate dispersion into calcium chloride solution, and for drug-chitosan-dispersion into tripolyphosphate solution, where beads were formed due to cross linking of alginate by calcium ions, and for chitosan with phosphoric ions. Preparation of beads by ionotropic gelation is based on the ability of various polysaccharides such as pectin, alginate, chitosan, and gellan to form a gel in the presence of multivalent ions. Beads can therefore be prepared simply by adding, drop wise, a dispersion of polysaccharide and material to be encapsulated into solution of multivalent ions. The contact of droplets with the ions results in instantaneous formation of spherical gel structures containing the material to be encapsulated uniformly dispersed throughout the polysaccharide matrix.

Alginate and chitosan micro beads were successfully prepared by ionotropic gelation method. The beads were coated with Eudragit S100 by spray coating in a conventional coating pan. The coated beads were found to be of spherical shape. Diameter of beads varies as shown in Table 2 for different formulations. The result indicates that as the amount of polymer (alginate and chitosan) increases, the size of beads proportionally increases.

**TABLE 2:** PHYSICO-CHEMICAL CHARACTERIZATION OF PREPARED HYDROGEL BEADS

Formulation	Diameter (mm)^a^,^b^	Entrapment Efficiency (%)^a^,^c^	Drug content (mg/100 mg beads)
A1	0.79±0.09	63.77±1.41%	17.45
A2	0.84±0.11	68.30±1.03%	23.04
A3	0.87±0.13	77.42±1.09%	25.55
A4	1.60±0.17	78.75± 1.27%	24.02
A5	0.91±0.13	81.58±1.81%	27.93
C1	0.55±0.09	55.21±1.52%	16.99
C2	0.67±0.13	65.47±1.23%	19.07
C3	0.76±0.11	72.55±1.02%	20.46
C4	0.89±0.07	79.60±1.43%	24.98

a Mean±SD

b =50 beads

c =3 trials.

Moreover, longer the cross linking time, lower the size of beads. The bead diameter decreased as the cross linking time was increased, because there is greater time for the cross linker to promote formation of cross links with chitosan molecules or alginate molecules and make a more compact mass. The longer the hardening time, the smoother and more spherical were the beads obtained. Coating thickness over drug bearing beads was obtained in terms of total weight gain (TWG) of beads after enteric coating with Eudragit S100 dispersion and their effect on *in vitro* drug release in simulated GI fluid was studied.

The effect of various formulations parameters on entrapment efficiency of prepared hydrogel beads are shown in Table 2. The entrapment efficiency of prepared beads varied from 63.77±1.41 to 81.58±1.81% for alginate beads and for chitosan beads it varies from 55.21±1.5 to 79.60±1.43%. Entrapment efficiency and drug content increased significantly with increasing polymer concentration (alginate or chitosan) as shown in Table 2. This is because the increase in polymer concentration resulted in formation of larger beads entrapping more drug. This result is well correlated with similar result reported earlier for alginate-chitosan beads of timolol maleate[16].

**Fig. 1:**
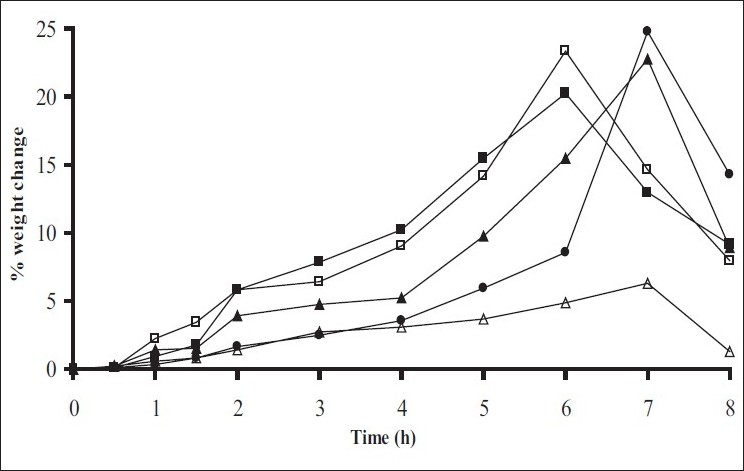
Swelling profile of alginate beads with Eudragit S100 coating (10% weight gain) A1(-■-), A2(-□-), A3(-▲-), A4(-Δ-), A5(-●-)

**Fig. 2:**
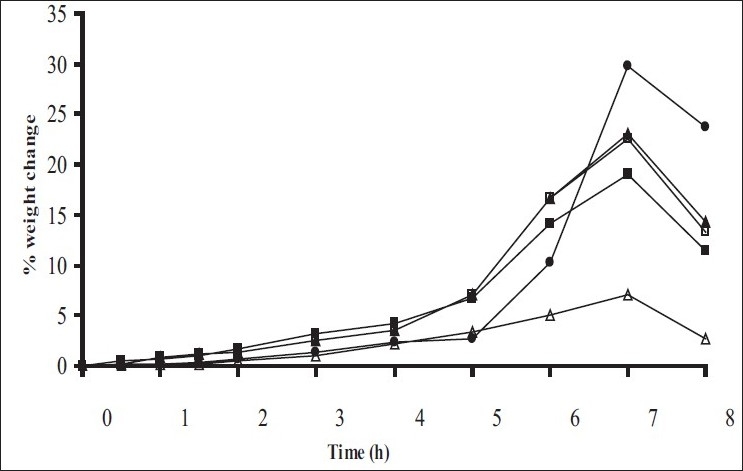
Swelling profile of Alginate beads with Eudragit S100 coating TWG 20% A1(-■-), A2(-□-), A3(-▲-), A4(-Δ-), A5(-●-)

**Fig. 3:**
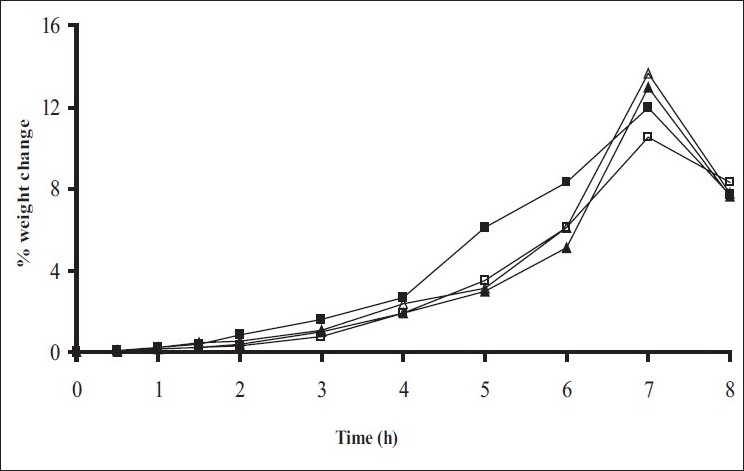
Swelling profile of Chitosan beads with Eudragit S100 coating TWG 10% C1(-■-), C2(-□-), C3(-▲-), C4(-Δ-)

**Fig. 4:**
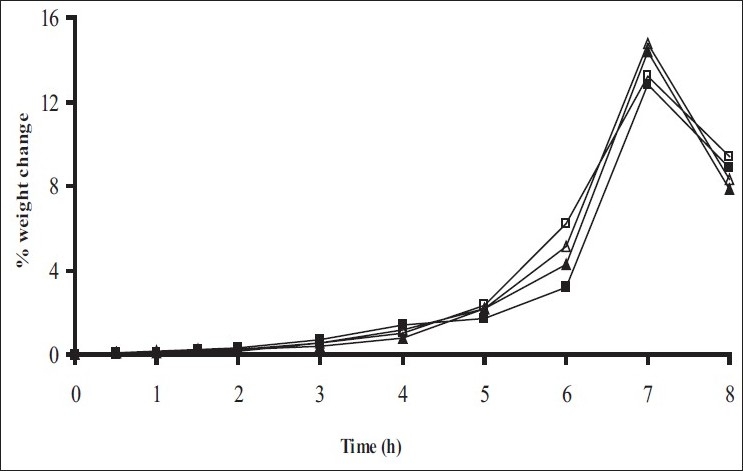
Swelling profile of Alginate beads with Eudragit S100 coating TWG 20% C1(-■-), C2(-□-), C3(-▲-), C4(-Δ-)

Swelling studies for coated beads was performed in three different pH (1.2, 6.8 and 7.4) simulating transition of beads in GIT. Swelling studies carried out shows that maximum swelling for all batches of beads is in simulated intestinal fluid (pH 7.4) as shown in figs. 1 to 4. The swelling of calcium alginate beads in phosphate buffer is related to Ca^2+^ and Na^+^ exchange. In initial phase Na^+^ ions present in phosphate buffer exchange with Ca^2+^ ions, bound to COO^-^ groups of mannuronic blocks. As a result, an electrostatic repulsion between negatively charged COO^-^ groups increases resulting in gel swelling. Exchanged Ca^2+^ ions precipitate in the form of insoluble calcium phosphate reflecting in slight turbidity of swelling medium. In the later phase of swelling, diffusion of Ca^2+^ from polyguluronate blocks cause loosening of tight egg-box structure and thus permit the penetration of additional amounts of media into the beads[17].

In case of chitosan beads, ionization of amine group on chitosan decreases with increasing pH proceeding to weaker cross linking density. At higher pH open porous structure with low density were also reported[18]. In case of ethylcellulose reinforced alginate beads the swelling was found to be to a lesser extent which is due to the hydrophobic nature of ethylcellulose.

In case of uncoated beads, nearly >70% of drug was released in initial 4 h of dissolution study. This situation is not acceptable for the drugs that are required to be released in colon. Therefore beads were coated with Eudragit S100 polymer to retard the release of drug until the pH reaches above 7.0.

**Fig. 5:**
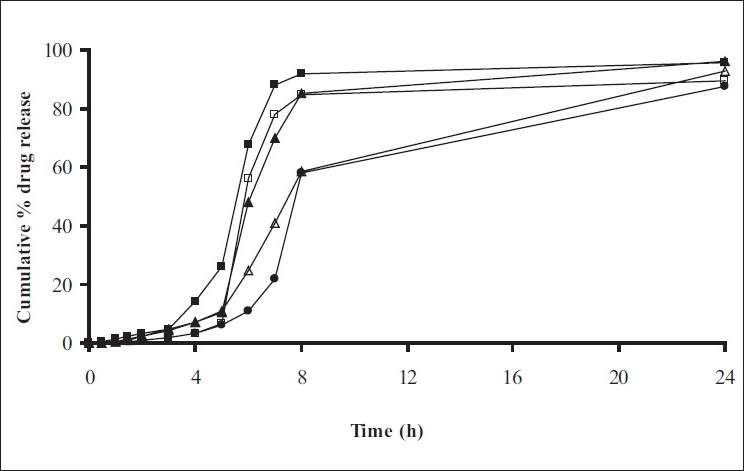
*In vitro* release profile of Alginate beads with Eudragit S100 coating TWG 10% A1(-■-), A2(-□-), A3(-▲-), A4(-Δ-), A5(-●-), (n=3)

**Fig. 6:**
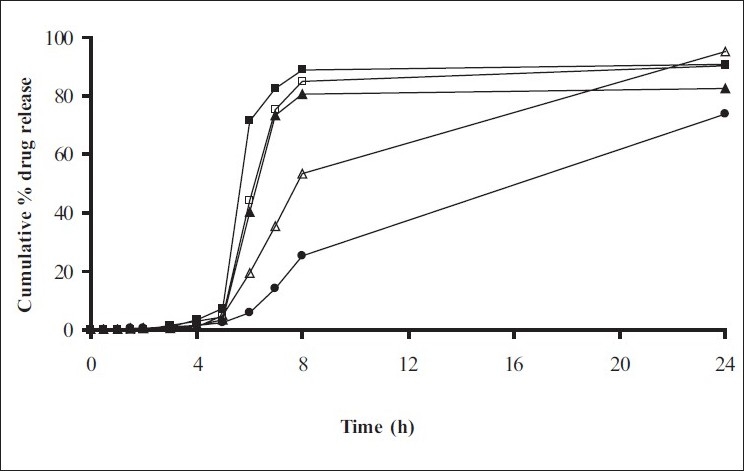
*In vitro* release profile of Alginate beads with Eudragit S100 coating TWG 20% A1(-■-), A2(-□-), A3(-▲-), A4(-Δ-), A5(-●-)

**Fig. 7:**
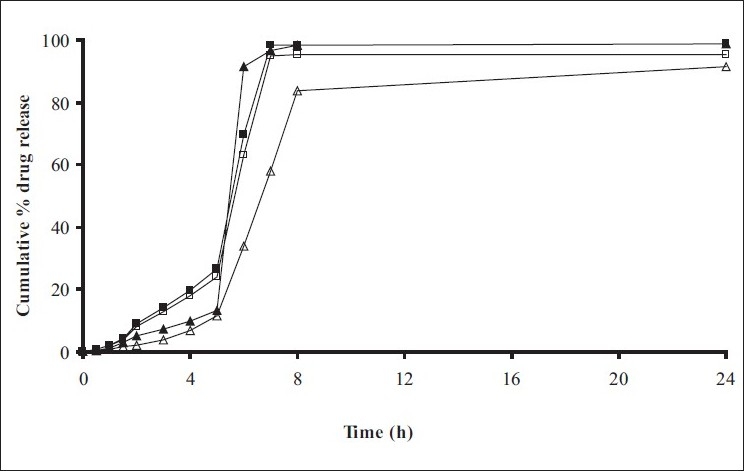
*In vitro* release profile of Chitosan beads with Eudragit S100 coating TWG 10% C1(-■-), C2(-□-), C3(-▲-), C4(-Δ-)

**Fig. 8:**
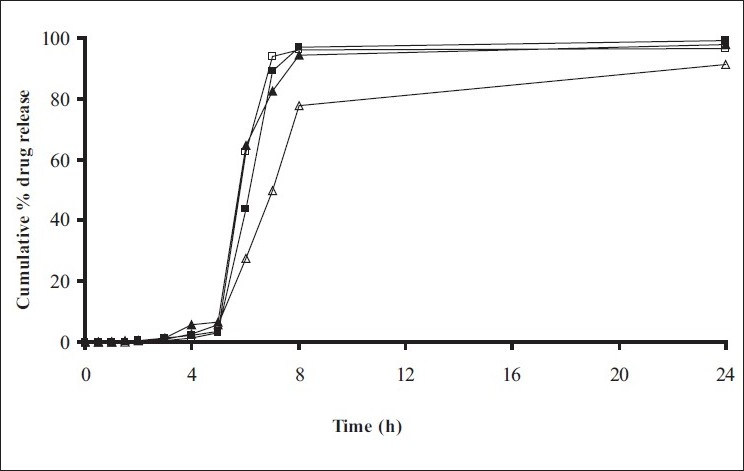
*In vitro* release profile of Chitosan beads with Eudragit S100 coating TWG 10% C1(-■-), C2(-□-), C3(-▲-), C4(-Δ-)

*In vitro* release studies were carried out in order to evaluate effect of Eudragit S100 coat with an objective to release most of drug in the colon. The initial release of TH from coated hydrogel beads was low. However, some part of drug (10 to 15%) was released in initial 2 h from the beads having TWG 10%. In case of TWG 20%, very less amount of drug was found to be released (3 to 4%) which is less as compared to drug release from TWG 10%. Figs. 5 to 8 show the drug release from various formulations in different simulated GI fluids.

In case of alginate beads (A1, A2, A3, A4, A5) having a coat of TWG 10%, at the end of 5 h the release was found to be 26.25±1.09, 6.80±1.81, 10.58±1.29, 11.07±1.59 and 6.07±2.35%, respectively. In case of beads having a coat of TWG 20%, Eudragit S100 coat release at end of 5 h was found to be 7.18±1.19, 4.51±1.23, 3.29±1.15, 4.23±1.29 and 2.66±1.89%, respectively. For chitosan beads (C1, C2, C3 and C4) having a coat of TWG 10% Eudragit S100 coat, release at end of 5 h was found to be 26.68±1.33, 18.06±1.28, 13.25±1.17 and 6.28±1.09%, respectively. In case of beads having a coat of TWG 20% Eudragit S100 coat release at the end of 5 h was found to be 3.59±1.63, 1.27±1.09, 6.73±1.13 and 2.54±1.47%, respectively.

However, >80% of drug was released when pH was gradually raised above 7.0. The result indicates that drug was protected completely from upper GIT condition by a coating of Eudragit S100, because Eudragit polymer contain carboxyl group that ionize in an environment where pH is greater then 7. As ionization take place, integrity of film was disturbed and drug is released. At pH 7.4, membrane coating get dissolved and beads were exposed to dissolution media following which the polymer matrix swells and erodes releasing entrapped drug.

Drug release data was plotted according to first order, Higuchi's and Korsemeyer-Peppas equation to know the release mechanisms. The formulations showed linearity with respect to first order (R^2^ = 0.8471–0.9591) as compared to zero order (R^2^ = 0.7022–0.8560) for formulations having TWG 10% coat thickness. Formulations having TWG 20% coat thickness also showed the linearity with respect to first order (R^2^ = 0.8833–0.9545) as compared to zero order (R^2^ = 0.7188–0.8090). Higuchi's equations for formulation having TWG 10% coat thickness ranges from (R^2^ = 0.7101–0.8278) and for formulation having TWG 20% coat thickness ranges from (R^2^ = 0.71936–0.7879) hence to confirm precisely the domination mechanism, the data was plotted according to Korsemeyer's equation. The resultant ‘n’ values was >1, for the all coated beads, indicating the super-case II transport mechanism.

Results of release studies indicate that Eudragit S100 coated hydrogel beads offer a high degree of protection from premature drug release in simulated upper GIT conditions. A well coat of Eudragit S100 delivers almost intact beads to colon[19], an environment rich in bacterial enzymes that degrade the polysaccharides and allow drug release to occur at desired site and can be a potential system for delivery of theophylline in cases of nocturnal asthma.
